# Development of anti-feline PD-1 antibody and its functional analysis

**DOI:** 10.1038/s41598-023-31543-6

**Published:** 2023-04-24

**Authors:** Shoma Nishibori, Mika K. Kaneko, Takayuki Nakagawa, Kazuo Nishigaki, Yukinari Kato, Masaya Igase, Takuya Mizuno

**Affiliations:** 1 Laboratory of Molecular Diagnostics and Therapeutics, Joint Faculty of Veterinary Medicine, Yamaguchi University, Yamaguchi, 753-8515 Japan; 2 Department of Molecular Pharmacology, Tohoku University Graduate School of Medicine, Sendai, 980-8575 Japan; 3 Laboratory of Veterinary Surgery, Graduate School of Agricultural and Life Sciences, The University of Tokyo, Tokyo, 113-8657 Japan; 4 Laboratory of Molecular Immunology and Infectious Disease, Joint Faculty of Veterinary Medicine, Yamaguchi University, Yamaguchi, 753-8515 Japan

**Keywords:** Tumour immunology, Cancer immunotherapy

## Abstract

Antibodies against immune checkpoint molecules restore T-cell function by inhibiting the binding of PD-1 and PD-L1 and have been shown to exert therapeutic effects in various human cancers. However, to date, no monoclonal antibody that recognizes feline PD-1 or PD-L1 has been reported, and there are many unknowns regarding the expression of immune checkpoint molecules and their potential as therapeutic targets in cats. Here we developed anti-feline PD-1 monoclonal antibody (1A1-2), and found that the monoclonal antibody against anti-canine PD-L1 (G11-6), which was previously developed in our laboratory, cross-reacted with feline PD-L1. Both antibodies inhibited the interaction of feline PD-1 and feline PD-L1 in vitro. These inhibitory monoclonal antibodies augmented the interferon-gamma (IFN-γ) production in activated feline peripheral blood lymphocytes (PBLs). Furthermore, for clinical application in cats, we generated a mouse-feline chimeric mAb by fusing the variable region of clone 1A1-2 with the constant region of feline IgG_1_ (ch-1A1-2). Ch-1A1-2 also augmented the IFN-γ production in activated feline PBLs. From this study, 1A1-2 is first anti-feline PD-1 monoclonal antibody with the ability to inhibit the interaction of feline PD-1 and PD-L1, and the chimeric antibody, ch-1A1-2 will be a beneficial therapeutic antibody for feline tumors.

## Introduction

Cancer is the leading cause of death in older companion animals, such as cats and dogs^1–3^. The most common types of tumors in cats include lymphoma, squamous cell carcinoma, mammary gland tumor, and soft tissue sarcoma^4,5^. Cancer treatment for companion animals depend on the tumor type, histologic grade, degree of tumor progression, individual's general condition, and the owner's financial situation. Established treatment options include surgery, radiation therapy, and chemotherapy^4,6^. However, currently available therapies are limited in curing many feline cancers, especially at advanced stages. Therefore, the development of novel therapies which are different from existing therapies that may be used in combination with current therapies is crucial.

Immunotherapy is a breakthrough in human cancer therapy and is a field of oncology that has recently made remarkable progress^7^. Among those, immune checkpoint molecules, such as cytotoxic T lymphocyte-associated antigen 4 (CTLA-4) and programmed cell death 1 (PD-1), have been receiving a lot of attention in the last decade. PD-1 molecules are expressed on the cell surface of CD4 + and CD8 + T cells, NK cells, and antigen-presenting cells such as macrophage and dendritic cells^8,9^. The interaction of PD-1 with programmed death-ligand 1 (PD-L1) downregulates the expression of certain antiapoptotic molecules, proinflammatory cytokines, and suppresses T-cell proliferation by inhibiting T-cell receptor signaling^10,11^. However, tumor cells express PD-L1 on their cell surface and bind to PD-1 on tumor-infiltrating lymphocytes, resulting in impaired cytokine production and cytotoxic activity against tumor cells^12,13^. Therefore, the inhibition of the PD-1/PD-L1 pathway can restore the effector functions of exhausted T cells, and monoclonal antibodies (mAbs) against PD-1 or PD-L1 that can enhance or restore T-cell effector functions have attracted much attention^14^. Based on these findings, several immune checkpoint inhibitors have been approved by the US Food and Drug Administration (FDA) for treatment and its therapeutic indications are expanding. Pembrolizumab and nivolumab, that were developed to target PD-1, were first approved by the FDA in 2014^15^, and since then, various mAbs against PD-1 and PD-L1 have been developed and numerous clinical trials have been conducted^16–21^. More recently, veterinary medicine has been focusing in the expression and functional analysis of these immune checkpoint molecules and their usefulness as therapeutic targets in animals^22^. In dogs, mAbs against PD-1/PD-L1 have been developed^23–25^, some of which have shown therapeutic efficacy in clinical trials^26–29^.

The feline PD-1 nucleotide sequence in cats was reported in 2010 and was found to have an amino acid sequence similar to that of humans and dogs^30^. It has also been reported that the protein expression of PD-1 and PD-L1 is elevated in blood lymphocytes from cats chronically infected with the feline immunodeficiency virus (FIV) compared that of uninfected cats^30^. Another study reported the elevation of PD-1 and PD-L1 mRNA expressions in peripheral blood mononuclear cells (PBMCs) obtained from cats with clinical signs associated with feline infectious peritonitis compared to healthy cats^31^. Furthermore, it has been reported that serum PD-1 and PD-L1 levels were significantly higher in cats with HER2-positive and triple negative (TN) normal-like mammary carcinomas^32^. PD-L1 expression in cancer cells was significantly higher in HER2-positive samples than in TN normal-like tumors^33^. We demonstrated the cross-reactivity of anti-human PD-L1 monoclonal antibody (clone 28-8) with feline PD-L1, and revealed that PD-L1 was expressed in macrophages in the spleen and lymph nodes of healthy cats and feline mast cell tumor tissues (in submission). However, to date, there are no mAbs against PD-1 and PD-L1 that can inhibit the interaction of these molecules. Thus, there are still many unknowns in terms of PD-1 and PD-L1 expression and the functional analysis of their interactions.

In this study, we have developed a novel mAb against feline PD-1. We also show that the anti-canine PD-L1 mAb, which was previously developed in our laboratory, cross-reacted with feline PD-L1. We also showed that these mAbs can inhibit the binding of feline PD-1/PD-L1 and restore the lymphocyte exhaustion. Furthermore, we generated a mouse-feline chimeric mAb against feline PD-1 for clinical use and analyzed their functions.

## Results

### Development of mAb against feline PD-1 and analysis of feline PD-1 expression in cell lines

**Figure 1:**
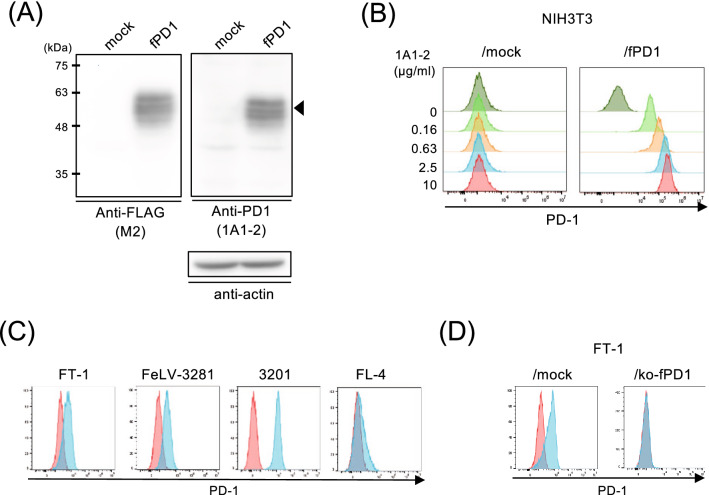
Specificity of anti-PD-1 mAb against feline PD-1 and analysis of PD-1 expression in feline cell lines. (**A**) Cell lysates from NIH3T3 (mock) and NIH3T3/fPD1 were used for western blotting. Proteins were separated by SDS-PAGE in two identical membranes, followed by western blotting using anti-FLAG antibody (M2) or anti-fPD1 mAb (1A1-2) in different membranes. Right blotting was reprobed with anti-β-actin antibody as the loading protein control. A representative result of 2 independent experiments is shown. Full images of western blotting at several different exposure time are shown in Supplementary Figure for Fig. 1A. (**B**) NIH3T3 or mock and NIH3T3/fPD1 or NIH3T3/fPD1 were stained with serially titrated anti-fPD-1 (1A1-2) mAb, followed by staining with secondary anti-mouse IgG-alexa647. (**C**) Flow cytometric analysis of feline PD-1 expression in feline cell lines. Cell lines were collected and stained with isotype control or anti-fPD-1 (1A1-2) mAb, followed by a secondary antibody. The red and blue histograms indicate the isotype control and 1A1-2 staining, respectively. (**D**) Flow cytometric analysis of feline PD-1 expression in feline PD-1-knocked out cell line, FT-1/ko-fPD1 cells. FT-1 cells, and FT-1/ko-fPD1 cells were collected and stained with the isotype control or anti-PD-1 (1A1-2) mAb, followed by a secondary antibody.

To obtain an mAb against feline PD-1, we first generated NIH3T3/fPD1 cells, which are NIH3T3 cells overexpressing feline PD-1 tagged with a FLAG. NIH3T3/fPD1 cells expressed a protein with a molecular weight of approximately 60 kDa determined by western blotting using the anti-FLAG antibody (Fig. 1A left panel). We immunized the mice with NIH3T3/fPD1 cells, and obtained 288 hybridoma clones, which were screened by flow cytometry using NIH3T3/fPD1 cells. Among these clones, clone 1A1-2 was found to bind to the feline PD-1 expressed on NIH3T3 cells (Fig. 1B). Clone 1A1-2 was a kappa light chain of the IgG_1_ subclass. The protein extracted from NIH3T3/fPD1 was detected by western blotting using 1A1-2, and the band showed the same molecular weight as that detected with anti-FLAG antibody (Fig. 1A right panel).

Next, the expression of PD-1 in feline cell lines was analyzed by flow cytometry using clone 1A1-2. As shown in Fig. 1C, PD-1 expression was detected in T-cell lymphoma cell lines (FT-1 and FeLV-3281), the thymic lymphoma cell line (3201), and the FIV-infected lymphoblastoid cell line (FL-4). PD-1 expression was not detected in the B-cell lymphoma cell line (MS4), PBMC-derived cell line (Fet-J), astrocyte cell line (G355), macrophage cell line (fcwf-4), and fibroblast cell line (CRFK), and all of mammary adenocarcinoma cell lines (FYMp, FKNp, FONp, FONm, and FMCm) (Table 1 and Supplement Figure). In addition, to confirm the specificity of clone 1A1-2 to feline PD-1, we generated a PD-1-knockout cell line, FT-1/ko-fPD1. Binding of clone 1A1-2 to FT-1 was lost after knockout of fPD-1 by flow cytometry (Fig. 1D).

**Table 1:** Summary of PD-1 and PD-L1 expressions in feline cell lines.

Cell lines	Origins	PD-1 expression	PD-L1 expression	
			IFN-γ (−)	IFN-γ ( +)
FT-1	T cell lymphoma (FeLV +)	+	−	−
FeLV-3281	T cell lymphoma (FeLV +)	+	+	+ +
MS4	B cell lymphoma	−	+	+
3201	Thymic lymphoma	+	−	−
FL-4	Lymphoblastoid (FIV +)	+	−	−
FeT-J	Lymphoblastoid	−	−	−
fcwf-4	Macrophage cells	−	−	+ (weak)
CRFK	Fibroblast	−	−	+ (weak)
G355	Astrocyte	−	−	−
FONp	Mammary adenosarcinoma	−	+	+ +
FYMp	Mammary adenosarcinoma	−	+	+ +
FKNp	Mammary adenosarcinoma	−	+	+ +
FONm	Mammary adenosarcinoma	−	−	−
FMCm	Mammary adenosarcinoma	−	−	+

### Cross-reactivity of anti-canine PD-L1 mAb to feline PD-L1 and analysis of feline PD-L1 expression in cell lines

**Figure 2:**
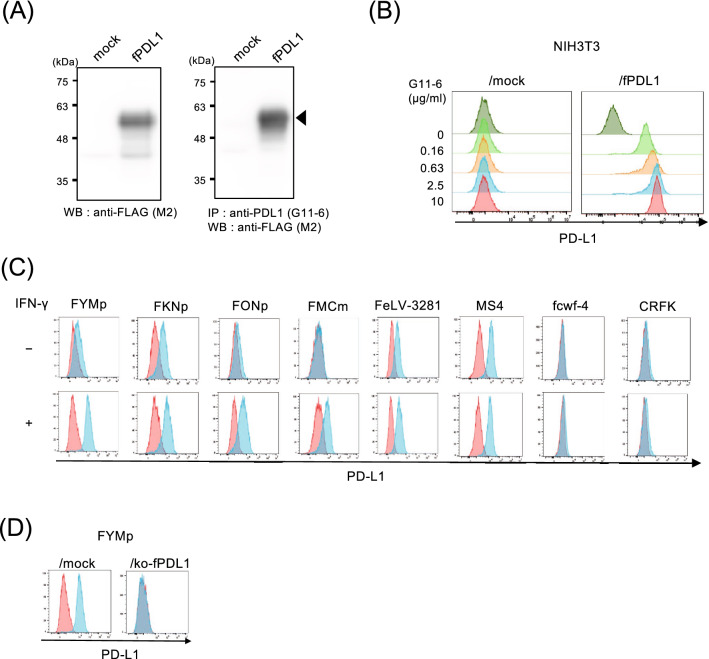
Cross-reactivity of anti-canine PD-L1 mAb with feline PD-L1 and analysis of PD-L1 expression in feline cell lines. (**A**) Cell lysates from NIH3T3 or mock and NIH3T3/fPDL1 or NIH3T3/fPDL1 were extracted and used for western blotting and immunoprecipitation. Western blotting was performed using anti-FLAG antibody (M2). For immunoprecipitation, cell lysates were immunoprecipitated with anti-canine PDL1 mAb (G11-6), and western blotting was performed using an anti-FLAG antibody (M2). Full images of western blotting at several different exposure time are shown in Supplementary Figure for Fig. 2A. (**B**) NIH3T3 or mock and NIH3T3/fPDL1 or NIH3T3/fPDL1 were stained with serially titrated mAb for anti-PD-L1 (G11-6) followed by staining with a secondary anti-rat IgG-DyLight649 antibody. (**C**) Flow cytometric analysis of feline PD-L1 expression in feline cell lines. Cell lines were treated with or without IFN-γ for 24 h. Cell lines were collected and stained with an isotype control or G11-6, followed by a secondary antibody. The red and blue histograms indicate the isotype control and G11-6 staining, respectively. (**D**) Flow cytometric analysis of feline PD-L1 expression in feline PD-L1-knocked out cell line, FYMp/ko-fPDL1 cells. Cell lines were treated with or without IFN-γ for 24 h. FYMp and FYMp/ko-fPDL1 cells were collected and stained with an isotype control or G11-6, followed by a secondary antibody.

We generated NIH3T3/fPDL1 cells, NIH3T3 cells overexpressing feline PD-L1 tagged with a FLAG and confirmed the expression of feline PD-L1 by western blotting using an anti-FLAG antibody, showing a band at a molecular weight of approximately 60 kDa (Fig. 2A left panel). We developed several anti-canine PD-L1 mAbs in our previous study^24^. Among these, anti-canine PD-L1 mAb, clone G11-6, was found to bind to feline PD-L1 by screening anti-canine PD-L1 mAbs by flow cytometry using NIH3T3/fPDL1 cells, and no other antibodies (Fig. 2B). However, the protein extracted from NIH3T3/fPDL1 could not be detected by western blotting using clone G11-6 (data not shown). Because clone G11-6 might recognize a conformational epitope of feline PD-L1, the protein extracted from NIH3T3/fPDL1 was subjected to immunoprecipitation with clone G11-6, followed by western blotting using the anti-FLAG antibody. As a result, a clear band at the same molecular weight as that detected with anti-FLAG antibody (Fig. 2A right panel).

To prove that clone G11-6 binds to endogenously expressed fPD-L1, the expression of PD-L1 in feline cell lines was analyzed by flow cytometry using clone G11-6. As shown in Fig. 2C, PD-L1 expression was detected in three out of five mammary adenocarcinoma cell lines (FYMp, FKNp, and FONp), the T-cell lymphoma cell line (FeLV-3281), and B-cell lymphoma cell line (MS4), but not in the macrophage (fcwf-4) and fibroblast (CRFK) cell lines. Expression of PD-L1 was induced in FYMp FKNp, FONp, and FMCm, and slightly in FeLV-3281, fcwf-4, and CRFK when cultured for 24 h with feline IFN-γ. As summarized in Table 2, neither PD-1 nor PD-L1 was expressed in PBMC-derived cell line (Fet-J), astrocyte cell line (G355), and one mammary adenocarcinoma cell line (FONm) (Supplement Figure). In addition, to further validate the specificity of the mAb to endogenous feline PD-L1, we generated feline PD-L1-knockout cell line, FYMp/ko-fPDL1. We confirmed that the binding of G11-6 was completely lost in FYMp/ko-fPDL1 cell line by flow cytometry (Fig. 2D). These results demonstrate the specificity of G11-6 for feline PD-L1, which was further used as an anti-feline PD-L1 antibody.

**Table 2:** The primers used for cloning and construction of plasmid vectors.

Primer name	For	Direction	Nucleotide sequences (5’ to 3’)
YTM1927	Cloning of full length feline PD-1	F	GTGGATCCCGTGGAGGAAGAGATTACGG (an underline indicates BamHI site)
YTM1928		R	TCGAATTCCGAGAGGAGAGCCAAGCTG (an underline indicates EcoRI site)
YTM1929	Cloning of full length feline PD-L1	F	TCGAATTCCAGCTCATTAGCGCGAGAAC (an underline indicates EcoRI site)
YTM1930		R	CCCTCGAGTTACGTCTCCTCAAATTGTAGATC (an underline indicates XhoI site)
YTM1934	Addition of FLAG tag to feline PD-1	R	GTCGATGTCATGATCTTTATAATC- GAGGGGCCAAGGGCA
YTM1935	Addition of FLAG tag to feline PD-L1	R	GTCGATGTCATGATCTTTATAATC- CGTCTCCTCAAATTG
YTM838	Amplification of FLAG tag	R	GTCGATGTCATGATCTTTATAATCGTCGATGTCATG
YTM2387	Knockout oligos of feline PD-1	F	CACCGTGGGGACCCCAACGGGCGCCC
YTM2388		R	AAACGGGCGCCCGTGGGGTCCCCAC
YTM2106	Knockout oligos of feline PD-L1	F	CACCGACCTGCTGCTGCAGCAGCTC
YTM2107		R	AAACCGAGCTGCTGCAGCAGCAGGTC
YTM1946	Expression vector for fPD1-hIg	F	CTTAGACTCCCCCTACAGG
YTM1947		R	CGGGATCCCTGGCCTTGGCCGGT
YTM1948	Expression vector for fPDL1-hIg	F	GTTTACGATCACAGTGTCC
YTM1949		R	CGAGATCTAGTCCTCTCATTTGCTGG

*F* forward, *R* reverse.

### Inhibition of PD-1/PD-L1 binding by mAbs against feline PD-1 and PD-L1

**Figure 3:**
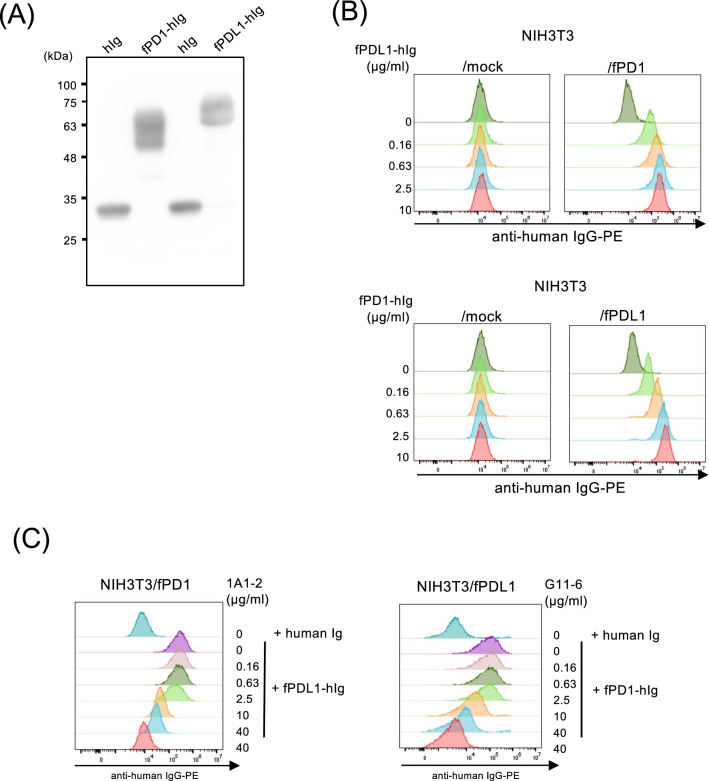
Inhibition of fPD-1 and fPD-L1 interaction by mAbs. (**A**) Western blotting of soluble fPD1-hIg and fPDL1-hIg fusion proteins. fPD1-hIg, fPDL1-hIg, and human IgG_2_-Fc (hIg) were purified from the supernatant of Expi-293F cells and submitted to western blotting with anti-human IgG-horseradish peroxidase (HRP). Full image of western blotting is shown in Supplementary Figure for Fig. 3A. (**B**) NIH3T3 or mock and NIH3T3/fPD1 and NIH3T3/fPD1 were stained with dose-titrated fPDL1-hIg fusion protein (left panel), or NIH3T3 or mock and NIH3T3/fPDL1 and NIH3T3/fPDL1 with fPD1-hIg (right panel) and followed by staining with a secondary anti-human IgG-PE antibody. (**C**) NIH3T3/fPD1 cells were preincubated with serially titrated anti-PD-1 (1A1-2) mAb, and, after washing, incubated with 1 μg/mL of fPDL1-hIg or human Ig (left panel). NIH3T3/fPDL1 cells were preincubated with anti-PD-L1 (G11-6) mAb, and, after washing, incubated with 5 μg/mL of fPD1-hIg or human Ig (right panel). Cells were then stained with a secondary antibody anti-human IgG-PE.

To verify whether these mAbs for feline PD-1 or PD-L1 inhibit the binding of feline PD-1 and PD-L1, the extracellular domains of feline PD-1 and PD-L1 were fused to the human IgG_2_ region to produce a soluble Fc fusion protein. The fusion proteins, fPD1-hIg and fPDL1-hIg were purified and detected at the expected molecular weight by western blotting using the anti-human IgG antibody (Fig. 3A). Furthermore, it was confirmed by flow cytometry that these fPD1-hIg and fPDL1-hIg proteins bound to NIH3T3/fPDL1 and NIH3T3/fPD1, respectively, not to the parental NIH3T3 (Fig. 3B). Preincubation of NIH3T3/fPD1 with anti-feline PD-1 mAb, clone 1A1-2, inhibited the binding of fPDL1-hIg in an antibody dose-dependent manner (Fig. 3C left panel). Similarly, anti-feline PD-L1 mAb, clone G11-6, inhibited the binding of fPD1-hIg to NIH3T3/fPDL1 (Fig. 3C right panel).

### Feline PD-1 and PD-L1 expressions in stimulated lymphocytes

**Figure 4:**
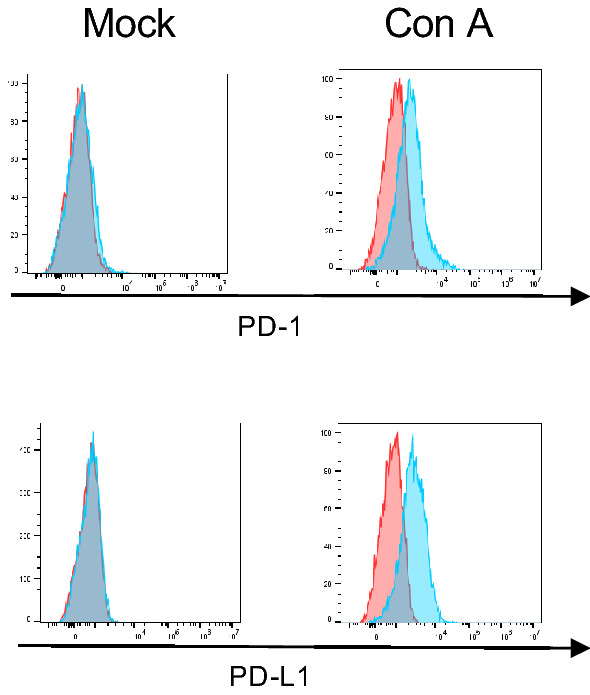
PD-1 and PD-L1 expression in stimulated feline lymphocytes. Peripheral blood lymphocytes were isolated from the peripheral blood and stimulated with 10 µg/mL of concanavalin A (Con A) for 48 h. After culture, the cells were collected and stained with anti-PD1 (1A1-2) mAb or anti-PD-L1 (G11-6) mAb, followed by anti-mouse IgG-alexa647 or anti-rat IgG-DyLight649 antibodies, respectively.

To investigate the expression of PD-1 and PD-L1 in feline immune cells, flow cytometry using anti-fPD-1 and fPD-L1 mAbs was performed on unstimulated and concanavalin A (Con A)-stimulated feline peripheral blood lymphocytes (PBLs). PD-1 and PD-L1 expression was not detected in unstimulated PBMC, but mitogenic stimulation with Con A for 48 h induced PD-1 and PD-L1 expression (Fig. 4).

### Production of IFN-γ stimulated by inhibition of PD-1/PD-L1 with mAbs

**Figure 5:**
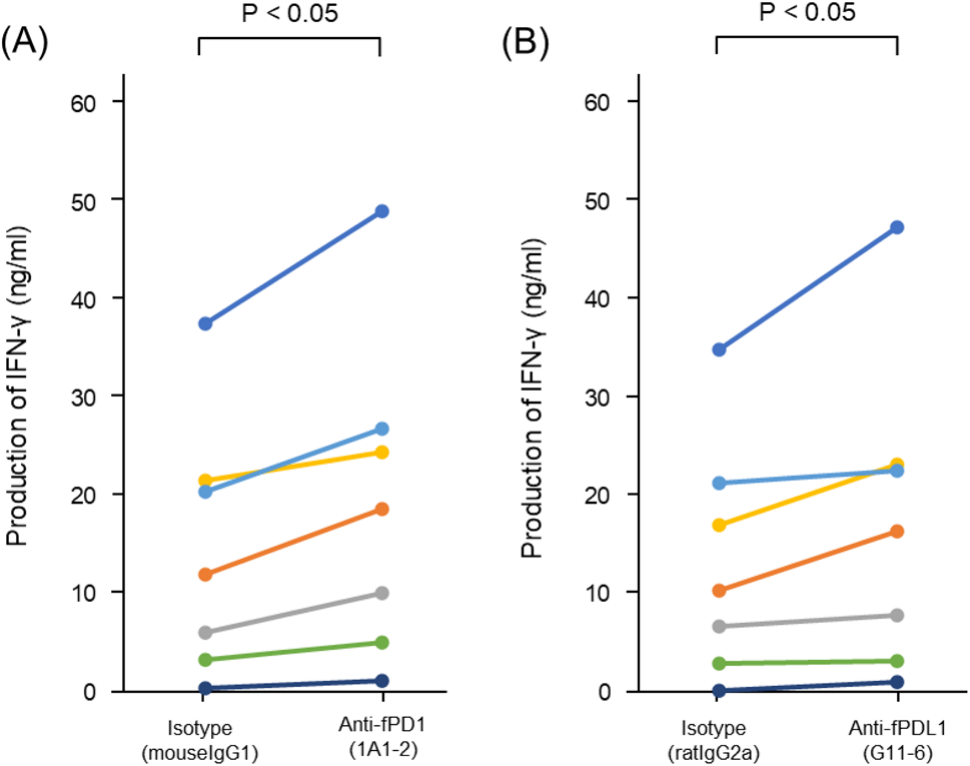
Augmentation of IFN-γ production in concanavalin A (Con A)-stimulated peripheral blood lymphocytes (PBLs) treatment with anti-PD-1 mAb or anti-PD-L1 mAb. PBLs were isolated from seven healthy cats and stimulated with 10 µg/mL of Con A for 48 h in the presence of mouse IgG_1_ isotype control or anti-fPD1 (1A1-2) mAb (**A**), rat IgG_2a_ isotype control or anti-fPDL1 (G11-6) mAb (**B**). After 48 h, the culture supernatant was collected and the amount of feline IFN-γ in the supernatants was measured in duplicate. Different color bars indicate different individuals.

To examine the effect of inhibiting fPD-1/PD-L1 binding in lymphocytes by mAbs, we measured the levels of IFN-γ released from the stimulated PBLs into the culture supernatant. As shown in Fig. 5A, PBLs collected from seven healthy cats were stimulated with 10 µg/mL of Con A and incubated with the isotype control or anti-fPD-1 mAb for 48 h. The IFN-γ production significantly increased in the presence of anti-fPD-1 mAb, 1A1-2 (p = 0.018). Similarly, the addition of anti-fPD-L1 mAb, G11-6, also significantly increased IFN-γ production (p = 0.018) (Fig. 5B).

### Generation of chimeric anti-feline PD-1 mAb and evaluation of in vitro activity

**Figure 6:**
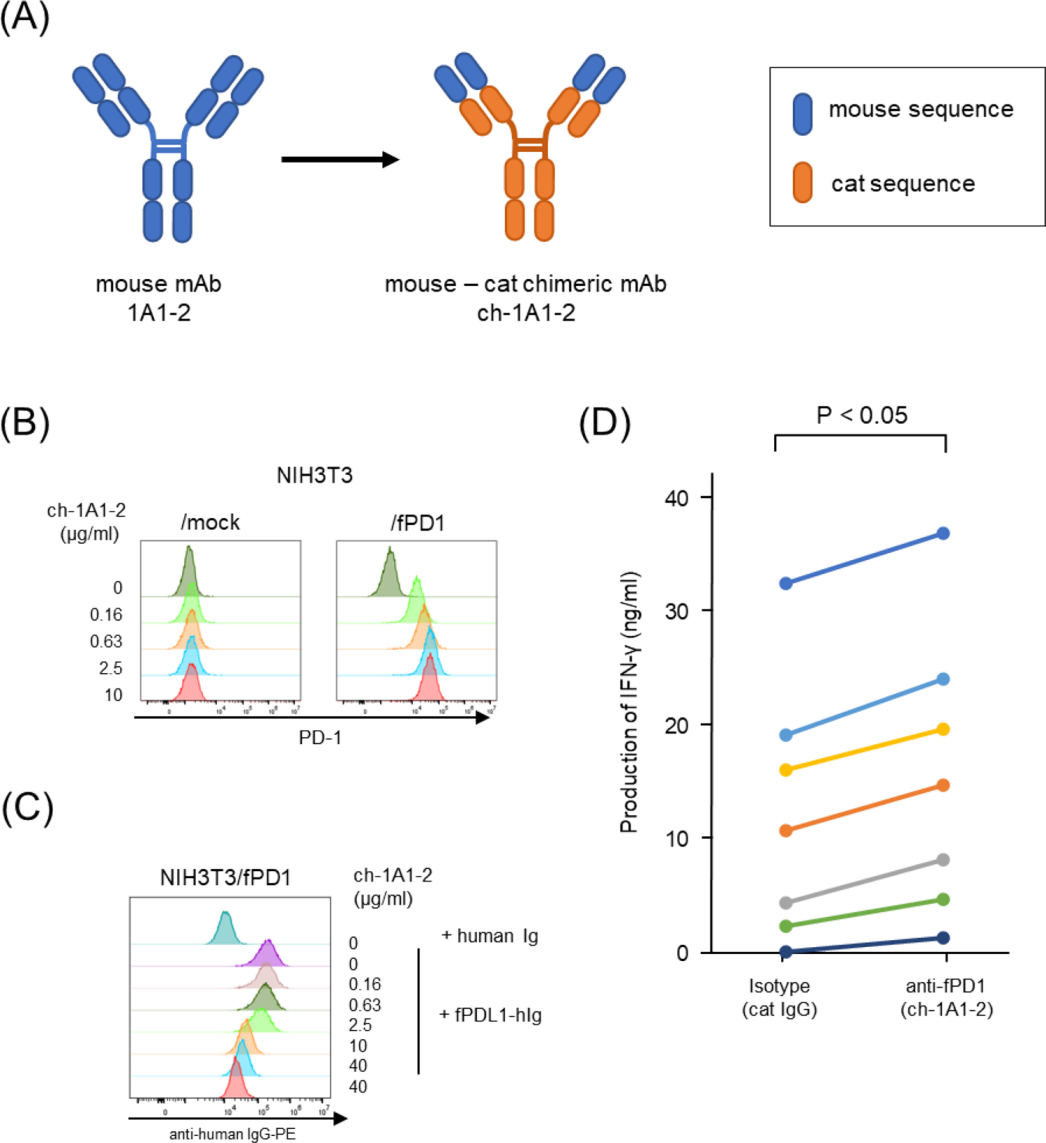
Generation of mouse-feline anti-PD-1 chimeric mAb and its functional analysis in vitro*.* (**A**) Schematic overview of mouse mAb, 1A1-2, and mouse-feline chimeric mAb, ch-1A1-2. The portions derived of mouse sequence are indicated by blue diagrams, and those of cat sequence are indicated by orange diagrams. (**B**) NIH3T3 or mock and NIH3T3/fPD1 or NIH3T3/fPD1 were stained with dose-titrated ch-1A1-2 followed by staining with a secondary anti-cat IgG-PE antibody. (**C**) NIH3T3/fPD1 cells were preincubated with dose-titrated ch-1A1-2, and, after washing, incubated with 1 μg/mL of fPDL1-hIg or human Ig. Cells were then stained with a secondary antibody anti-human IgG-PE. (**D**) PBLs were isolated from seven healthy cats and stimulated with 10 µg/mL of concanavalin A (Con A) for 48 h in the presence of a cat IgG isotype control or anti-fPD1 (ch-1A1-2). After 48 h, the culture supernatant was collected and the amount of feline IFN-γ was measured in duplicate. Different color bars indicate different individuals.

To reduce the immunogenicity of anti-fPD-1 mAb for clinical application, we generated a mouse-feline chimeric mAb (ch-1A1-2) by fusing the variable region of clone 1A1-2 with the constant region of feline IgG_1_ (Fig. 6A). We confirmed that ch-1A1-2 specifically bound to NIH3T3/fPD1 by flow cytometry (Fig. 6B). We also confirmed that preincubation of ch-1A1-2 with NIH3T3/fPD1 inhibited fPDL1-hIg binding (Fig. 6C). Furthermore, PBLs from seven healthy cats were stimulated with 10 µg/mL of Con A in the presence of the isotype control or ch-1A1-2, and IFN-γ production significantly increased in the presence of ch-1A1-2 (p = 0.018) (Fig. 6D).

## Discussion

In this study, we developed a novel mAb against feline PD-1 (clone 1A1-2) and showed that the anti-canine PD-L1 mAb (clone G11-6) developed in our previous study^24^, cross-reacted with feline PD-L1. It is noteworthy that this is the first report about mAbs against feline PD-1 and PD-L1, which demonstrated specificity to both molecules and inhibited the interaction between these molecules. The specificities of these mAbs are highly reliable, as they have been shown to bind to cell lines overexpressing feline PD-1 or PD-L1, and react with endogenous feline PD molecules using feline cell lines, mitogen stimulated lymphocytes, and cell lines in which PD molecules have been knocked out. However, although the predicted molecular weights of feline PD-1 and PD-L1 are 30 kDa and 33.4 kDa, respectively, western blotting using 1A1-2 and immunoprecipitation using G11-6 results showed that the molecular weights of feline PD-1 and PD-L1 are approximately 60 kDa. According to previous reports, mouse PD-1 and PD-L1^34,35^ and human PD-1 and PD-L1 were glycosylated^36,37^. In addition, western blotting of canine PD-1 and PD-L1 also showed that the bands had a molecular weight that was larger than expected^24^, indicating that feline PD-1 and PD-L1 are similarly glycosylated. Especially, several bands with different molecular weight sizes shown in western blotting of feline PD-1 (Fig. 1A) suggested that feline PD-1 was modified with various glycosylation, as it has multiple glycosylation sites as human PD-1^38^.

In this study, the expressions of PD-1 and PD-L1 were not observed in unstimulated PBLs, and the induced levels of PD-1 expression was weak even when stimulated with Con A. One possible reason why PD-1 expression in feline lymphocytes was not well induced by even stimulation with a mitogen is that 1A1-2 may not be sufficiently sensitive to detect PD-1 in flow cytometry. However, the cause of this is not clear, as flow cytometric analysis with 1A1-2 in feline cell lines was able to detect sufficient PD-1 expression. Previous experiments have reported that PD-1 and PD-L1 expressed in feline PBMCs were increased by mitogen stimulation and irradiation^30^. PD-1 expression was greatly increased following mitogen stimulation in canine PBMCs^19,32^. In addition, the expression of PD-1 in stimulated PBMCs is a phenomenon commonly seen in mice, humans, and bovine^34,39,40^, and the result of our experiment are inconsistent with these reports. Generally, Con A is a strong T-cells stimulant, however, T-cell stimulation with Con A may not be a proper stimulant to induce the expression of PD-1 in cats.

Notably, the present study is the first report to evaluate the restoration of lymphocyte function in cats when inhibited with PD-1 and PD-L1 binding by measuring the levels of IFN-γ. Both anti-PD-1 and anti-PD-L1 inhibitory mAbs significantly increased in IFN-γ production, but these increments in IFN-γ production were variable between individuals. In a previous study in dogs, differences in the effects of anti-PD-1 and anti-PD-L1 inhibitory mAbs between individuals were also observed^24,25^, suggesting that the differences of PD-1 and PD-L1 expression in stimulated lymphocytes may have influenced the results. This study showed a significant increase of IFN-γ production stimulated by anti-PD-1 or anti-PD-L1 mAbs, but the trend was observed more strongly when anti-PD-1 antibody was added. We cannot directly compare the ability of these clones to inhibit PD-1 and PD-L1 binding only from this study, but it may be caused by the difference of inhibitory effects of those antibodies. It may also be due to the several binding partners for both PD-1 and PD-L1. In addition to binding to PD-L1, PD-1 has been shown to bind to PD-L2, resulting in the inhibition of T-cell receptor-mediated proliferation and cytokine production^41^. PD-L1 has been shown to bind to CD80 in addition to PD-1, and CD80 has been shown to limit the PD-1 co-inhibitory signal, while promoting CD28-mediated co-stimulation^42^. These mechanisms have not been analyzed in cats to date, and therefore, further research is required for a deeper understanding of PD-1/PD-L1 interactions in cats.

Few therapeutic feline chimeric or felinized antibodies have been reported to date, and the only commercially available one, called Frunevetmab, is a felinized anti-nerve growth factor (NGF) mAb, for alleviation of osteoarthritis pain in cats. These antibodies were intended to neutralize feline herpesvirus, calicivirus, and NGF, and the feline chimeric antibodies were produced through the fusion of the feline IgG_1_ constant region with the variable region of the respective antibodies^43,44^. The mouse-feline chimeric mAb against feline PD-1 made in this study, ch-1A1-2, was also produced by fusing the variable region with the constant region of feline IgG_1_. The ch-1A1-2 was shown to have the same inhibitory ability as 1A1-2 by flow cytometry using human Ig fusion protein. Furthermore, the results of IFN-γ assay using this chimeric mAb showed that the addition of ch-1A1-2 significantly increased the production of IFN-γ, suggesting that it can restore lymphocyte exhaustion. Previous reports have shown that IgG_2_ occurs infrequently in healthy cats (~ 2%) and the majority produce IgG_1_^45^. However, IgG_1_ has a higher affinity for fFcγRI and fFcγRIII than IgG_2_^45^, and thus antibody dependent cellular cytotoxicity (ADCC) may be induced when ch-1A1-2 is administered in vivo. In this experiment, we used PBLs, which included B and NK cells, and a small amounts of remaining monocytes. The reason for the confirmed inhibitory effect of ch-1A1-2 is due to the removal of monocytes in this experiment. However, there has been no report to date that has evaluated the ADCC activity of feline IgG_1_, and it is necessary to investigate whether ch-1A1-2 has ADCC activity in in vitro and in vivo experiments in the future.

In conclusion, we have succeeded in obtaining mAbs that specifically bind to feline PD-1 or PD-L1 and have demonstrated that these mAbs inhibited PD-1/ PD-L1 binding. We also showed that the inhibition of PD-1/PD-L1 binding in lymphocytes by the mAbs restored lymphocyte exhaustion. Furthermore, we produced a functional mouse-feline chimeric mAb against feline PD-1 for clinical application. These results suggest that the inhibition of PD-1/PD-L1 binding in the tumor microenvironment may have anti-tumor effects in cats, and provide the fundamental tools for the establishment of immune checkpoint molecular inhibition therapy in cats as well as in humans and dogs. In the future, for making this antibody finally commercialized, safety and antibody pharmacokinetic studies using healthy cats are necessary, and it is expected that the final step of confirming safety and anti-tumor efficacy in cancer-bearing cats will be conducted.

## Methods

All animal procedures were approved by the Yamaguchi University Ethics Committee (ID#02-12) and the Zenoaq Ethics Committee (ID#22051). All experiments were conducted in accordance with relevant guidelines and regulations, including the Animal Research: Reporting of In Vivo Experiments (ARRIVE) guidelines.

### Cell lines

The D10 complete medium (Dulbecco's Modified Eagle Medium (DMEM) supplemented with 10% fetal bovine serum (FBS), 100 units/mL penicillin, 100 µg/mL streptomycin, and 55 µM 2-mercaptoethanol) was used to maintain the human kidney cell line, HEK293T, packaging cell line, PLAT-E^46^, mouse fibroblast cell line, NIH3T3^47^, feline fibroblast cell line, Crandell-Rees Feline Kidney Cell (CRFK)^48^, feline macrophage cell line, fcwf-4^49^, feline T-cell lymphoma cell line, FeLV-3201, feline thymic lymphoma cell line, 3201^50^, and feline astrocyte cell line, G355^51^. Only the FeLV-3281 cell line was used in D10 medium without 2-mercaptoethanol. Additionally, the feline T-cell lymphoma cell line, FT-1^52^, feline B-cell lymphoma cell line, MS4^53^, feline lymphoblastoid cell line, FL-4^54^, the cell line from peripheral blood mononuclear cells, Fet-J^55^, and feline mammary gland tumor cell lines, FONp, FYMp, FONm, FKNp, and FMCm^56^, were kept in R10 complete medium (RPMI-1640 supplemented with 10% FBS, 100 units/mL penicillin, 100 µg/mL streptomycin, and 55 µM 2-mercaptoethanol). These cell lines were cultured at 37 °C in a humid incubator with 5% CO_2_. The NIH3T3 cells were obtained from the Institute of Development, Aging, and Cancer at Tohoku University. The FeLV-3281, MS4, Fet-J, G355 cell lines were kindly provided by Dr. Kazuo Nishigaki (Yamaguchi University). Furthermore, Dr. Hajime Tsujimoto generously donated CRFK, fcwf-4, FT-1, and FL-4 (The University of Tokyo). All feline mammary gland tumor cell lines were kindly provided by Dr. Takayuki Nakagawa (The University of Tokyo).

### Constructions of the mammalian expression vectors and the human immunoglobulin fusion protein vectors

All primers used for the molecular recombination are detailed in Table 2. Feline PD-1 and PD-L1 were cloned using the PCR primers, YTM1927 and YTM1928, YTM1929 and YTM1930, with the cDNAs of KO-1 cell line^57^ and FL-4 cell line as a template, respectively. Both amplified products were cut with BamHI and EcoRI, and EcoRI and XhoI and ligated into the BamHI and EcoRI sites of pMx-IP (pMx-IP-fPD1-K#9) and EcoRI and XhoI sites of pMx-IP (pMx-IP-fPDL1-K#14). To add a FLAG-tag, PCR was performed with YTM1927 and YTM1934, and YTM1929 and YTM1935 with pMx-IP-fPD1-K#9 and pMx-IP-fPDL1-K#4 as a template, followed by another PCR using the amplified product with primers YTM1927 and YTM838 and primers YTM1929 and YTM838, and was then inserted into the pBluescript SK (-) vector and pANT vector (Nippon genetics) to prepare pBS-fPD1-FL#4 and pANT-fPDL1-FL#1, respectively. The fragments of pBS-fPD1-FL#4 and pANT-fPDL1-FL#1 cut with BglII and SmaI were inserted into the BamHI-SnaBI site of pMx-IP, resulting in pMx-IP-fPD1-FL#1 and pMx-IP-fPDL1-FL#9. The nucleotide sequence analysis of each plasmid was performed by the DNA Core Facility of the Center for Gene Research, Yamaguchi University, and compared with the nucleotide sequences of feline PD-1 and PD-L1 registered in the public database (NM_001145510.1^30^ and LC735019^58^, respectively).

To prepare the knockout vector for feline PD-1 and PD-L1, YTM2387 and YTM2388, and YTM2106 and YTM2107 were annealed and put into lentiCRISPRv2 to make lentiCRISPR-fPD1#2 and lentiCRISPR-fPDL1-2#1, respectively. lentiCRISPRv2 was a gift from Dr. Feng Zhang (Addgene plasmid #52961; http//n2t.net/addgene:52961; RRID: Addgene_52961).

Vectors expressing fPD-1 or fPD-L1 with the Ig fusion protein were constructed. To construct the expression vectors, PCR was performed with the primers YTM1946 and YTM1947 or YTM1948 and YTM1949, using pBS-fPD1-FL#4 or pANT-fPDL1#1 as the templates. The fPD-1 and fPD-L1 PCR products were cut with BamHI or BglII, and cloned into the EcoRV and BglII restriction sites of the pFUSE-hIgG_2_-Fc2 vector (InvivoGen, California, US), resulting in pFUSE-fPD1-hIg#1 and pFUSE-cPDL1-hIg#1, respectively.

### Establishment of stably transduced cells

NIH3T3 cells that stably express fPD-1 and fPD-L1 were established using retroviral transduction. Briefly, PLAT-E cells (7.5 × 10^5^ cells) were seeded in a 6-well dish, one day before transfection. Cell transfection was performed using the PEI Max method. A mixture of 1.25 μg of pMx-IP-fPD1-FL#1 or pMx-IP-fPDL1-FL#9 was incubated with 5 μL of 1 mg/mL polyethylenimine (PEI) “Max” (Polysciences Inc., Pennsylvania, USA) in 62.5 μL of OPTI-MEM for 15 min at room temperature, then added to the cell media. The medium was changed to new DMEM containing 10% FBS 24 h after transfection. After a further 24 h, the supernatant was collected from each transfected culture and used for viral transduction into NIH3T3 cells as previous described^59^. The transduced cells were then cultured in the presence of 10 μg/mL of puromycin (Sigma-Aldrich Japan K.K., Tokyo, Japan) to obtain stably transduced cells (NIH3T3/fPD1 and NIH3T3/fPDL1).

Feline PD-1 was knocked out in FT-1 cells (FT-1/ko-fPD1), and feline PD-L1 was knocked out in FYMp cells (FYMpko-fPDL1) using lentivirus transduction. Briefly, HEK293T cells (7.5 × 10^5^ cells) were seeded in a 6 well plate, one day before transfection. A mixture of 0.375 µg of lentiCRISPR-fPD1#2 or lentiCRISPR-fPDL1-2#1 was incubated with 0.5 µg of p8.9QV, 0.375 µg of pCVSVG, and 5 µL of 1 µg/mL PEI Max (Polysciences Inc.) in 62.5 µL of OPTI-MEM for 15 min at room temperature, and was added to the cell media. Subsequent steps are the same as above, but 1.5 µg/mL or 2 µg/mL of puromycin (Sigma-Aldrich) was used for FT-1/ko-fPD1 and FYMp/ko-fPDL1 selection, respectively.

### Production of a mouse mAb against feline PD-1

A panel of antibodies for fPD-1 was obtained by immunization with NIH3T3/fPD1 as described in a previous report^60^. In brief, 1 × 10^7^ of each of the transduced cells were prepared in 500 μL phosphate buffered saline (PBS) and emulsified with an equal amount of Titer Max Gold (CytRx, California, USA). The emulsified cells were injected intracutaneously into the hind footpads of 5-week-old BALB/cCrSlc mice (Japan SLC, Hamamatsu, Japan). Immunization was performed once a week, for 4 times in total. Ten days after the last immunization, popliteal lymph node cells were collected and fused with P3U1 cells, using the ClonaCell-HY kit (Veritas, Tokyo, Japan). Hybridomas producing antibodies against fPD-1 were identified by cell ELISA and were subsequently cloned.

Supernatants from the hybridoma cultures producing anti-feline PD-1 antibody, 1A1-2, or anti-canine PD-L1 antibody, G11-6, were collected, and purified using MabSelect SuRe (Cytiva) column with AKTA start (Cytiva).

### Production and purification of immunoglobulin fusion proteins

The pFUSE-fPD1-hIg#1 and pFUSE-fPDL1-hIg#1 were transduced into Expi293 F cells using the Expi293 expression system (Thermo Fisher Scientific, Waltham, MA, USA) according to manufactures’ instruction. fPD1-hIg and fPD-L1-hIg were collected and purified using a HiTrap Protein A HP column (Cytiva) and desalted using a PD10 column (Cytiva)^24^.

### Western blotting

Cells were collected, washed once with cold PBS, and lysed at 4 °C for 15 min in lysis buffer [1% NP40, 10 mM Tris–HCl (pH 7.5), 150 mM NaCl, 1 mM EDTA, protease inhibitor cocktails (Nacalai Tesque, Kyoto, Japan), 1 mM Na_3_VO_4_, and 50 mM NaF], they were then centrifuged at 15,000 rpm for 15 min at 4 °C. The supernatant was collected, and the amount of protein in the cell lysate was measured using a TaKaRa BCA Protein Assay Kit (TaKaRa Bio, Shiga, Japan) according to the manufacturer’s recommendations. The samples were separated on a 10% acrylamide gel before proteins were blotted onto a PVDF membrane (Merck, Darmstadt, Germany). After blotting, the membrane was blocked with blocking buffer (Tris-buffered saline with 0.05% Tween 20 and 5% skim milk) for 1 h at room temperature. The membrane was incubated with a primary antibody, mouse monoclonal anti-FLAG antibody (M2; Sigma-Aldrich; dilution 1:1000), an anti-fPD1 antibody in TBS-T with 0.5% skim milk at 4 °C overnight. The membrane was washed 3 times with TBS-T for 10 min at a time and then incubated with horseradish peroxidase (HRP)-conjugated goat anti-mouse IgG secondary antibody (Biolegend Japan, Tokyo, Japan; dilution 1:5000) for 1 h. The membrane was imaged with an Amersham ImageQuant 800 after being washed a further three times with TBS-T for 10 min (Cytiva).

### Immunoprecipitation

Cells were collected and washed once with cold PBS. The cells were lysed for 30 min at 4 °C in 250 µL of lysis buffer [1% NP40, 10 mM Tris–HCl (pH 7.5), 150 mM NaCl, 1 mM EDTA, protease inhibitor cocktails (Nacalai Tesque), 1 mM Na_3_VO_4_, and 50 mM NaF], centrifuged at 12,000 × g for 5 min at 4 °C, and the supernatant was collected. The cell lysates were precleared with 10 µL of protein A/G agarose (Santa Cruz Biotechnology, Dallas, Texas, USA) for 1 h at 4 °C with rotation. One microgram of anti-rat PD-L1 antibody (G11-6) was also incubated with 10 µL protein A/G agarose for 1 h at 4 °C with rotation. The precleared lysates and beads with antibodies were mixed at 4 °C overnight with rotation. The immunoprecipitants were washed five times with PBS and dissolved with 1 × loading dye. Western blotting was then performed as described above using with a primary antibody, mouse monoclonal anti-FLAG antibody (M2; Sigma-Aldrich; dilution 1:1000).

### Production of mouse-feline chimeric mAb against fPD-1

To obtain the recombinant antibody, we cloned the cDNA heavy chain variable region of 1A1-2 and cDNA constant region of feline IgG_1_ into the pCAGGS-MCS vector, along with the cDNA light chain variable region of 1A1-2 and cDNA constant region of the feline kappa light chain into the pCAGGS-MCS vector. Both expression plasmids were transfected into Expi-293F cells using Expi293 expression system and the produced proteins were purified as described in the section “Production and purification of immunoglobulin fusion proteins.” ChromPure Cat IgG, whole molecule, was purchased from Jackson ImmunoResearch Inc. (West Grove, PA, USA).

### Flow cytometry

Cell staining by flow cytometry was performed as described in a previous report^59^. Cells were collected and washed with flow cytometry buffer (PBS with 2% FBS and 0.1% NaN_3_). Cell surface expression of PD-1 was detected by incubation with 10 μg/mL of anti-fPD-1 or an isotype control (mouse IgG_1_ kappa isotype control; Thermo Fisher Scientific) followed by incubation with alexa-647 conjugated anti-mouse IgG antibody (Jackson ImmunoResearch Laboratories, Pennsylvania, USA; dilution 1:500), or incubation with anti-PD-1 chimeric antibody (ch-1A1-2) or an isotype control (cat IgG isotype control; Jackson ImmunoResearch Laboratories) followed by incubation with HiLyte Fluor 647-conjugated anti-cat IgG antibody (dilution 1:2000), which was made by labelling of anti-cat IgG antibody (Jackson ImmunoResearch Laboratories) with Ab-10 Rpid HiLyte Fluor 647 Labelling Kit (DOJINDO MOLECULAR TECHNOLOGIES, INC., Kumamoto, Japna) in our lab. Cell surface expression of PD-L1 was detected by incubation with 10 μg/mL anti-cPD-L1 or an isotype control (rat IgG_2a_ kappa isotype control; Biolegend Japan) followed by incubation with DyLight649-conjugated anti-rat IgG antibody (Biolegend Japan; dilution 1:1000). For the PD-1 and PD-L1 binding assay, soluble fusion proteins of fPD1-hIg (5 μg/mL)or fPDL1-hIg (1 μg/mL) were used to detect the cell surface expression of fPD-L1 or PD-1, respectively, followed by staining with PE-conjugated gamma chain specific anti-human IgG antibody (Jackson ImmunoResearch Laboratories; dilution 1:500) as a secondary antibody. Cells were incubated with propidium iodide immediately before flow cytometric analysis. Results derived from CytoFLEX (Beckman Coulter, California, USA) were analyzed using FlowJo software (Treestar, San Carlos, CA, USA). For IFN-γ-induced PD-L1 expression analysis, each cell line was incubated with 10 ng/mL of feline IFN-γ (R&D Systems, Minneapolis, MN, USA) for 24 h and collected for staining.

### Primary cell culture

PBMCs from specific pathogen-free cats (one 3 year olds, four 4 year old and two 6 year old), which are kept in our veterinary teaching hospital or NIPPON ZENYAKU KOGYO Co., Ltd. as a blood donor, were separated by density gradient centrifugation using Lymphoprep (Axis-shield, Oslo, Norway). For PD-1 and PD-L1 expression analysis, the isolated PBMCs were cultured in R10 for 1.5 h to attach monocytes onto the culture dish, and only the lymphocytes in the supernatant were collected. The collected PBLs were cultured in R10 medium in the absence (unstimulated control) or presence of 10 µg/mL of Con A at 37 °C for 48 h. The cells were stained with antibodies for flow cytometric analysis.

For the IFN-γ production assay, PBLs are collected by incubating PBMCs and attaching monocytes as in the flow cytometry analysis, 2 × 10^5^ PBLs of were cultured in R10 medium in the presence of 10 µg/mL of Con A with 10 µg/mL of mouse IgG_1_ isotype control (Biolegend) or anti-PD-1 antibody (1A1-2), or rat IgG_2a_ isotype control (Biolegend) or anti-PD-L1 antibody (G11-6), or cat IgG isotype control (Jackson ImmunoResearch Laboratories) or anti-PD-1 chimeric antibody (ch-1A1-2) at 37 °C for 48 h. The cell supernatants were collected, and the amount of IFN-γ was measured with DuoSet ELISA Development System feline IFN-γ (R&D systems, Inc.).

### Statistical analysis

Data sets of the two treatment groups for IFN-γ quantitation were compared using Wilcoxon signed-rank sum test. The level of statistical significance was set at p < 0.05.

## Supplementary Information

Supplementary Figures.

## Data Availability

The data that support the findings of this study are available from the corresponding author upon reasonable request.

## References

[1] Cannon CM (2015). Cats, cancer and comparative oncology. Vet. Sci..

[2] Merlo DF (2008). Cancer incidence in pet dogs: Findings of the Animal Tumor Registry of Genoa, Italy. J. Vet. Intern. Med..

[3] Fleming JM, Creevy KE, Promislow DEL (2011). Mortality in north american dogs from 1984 to 2004: An investigation into age-, size-, and breed-related causes of death. J. Vet. Intern. Med..

[4] Blackwood L (2013). Cats with cancer: Where to start. J. Feline Med. Surg..

[5] Graf R (2016). Swiss Feline Cancer Registry 1965–2008: The influence of sex, breed and age on tumour types and tumour locations. J. Comp. Pathol..

[6] Biller B (2016). 2016 AAHA oncology guidelines for dogs and cats. J. Am. Anim. Hosp. Assoc..

[7] He X, Xu C (2020). Immune checkpoint signaling and cancer immunotherapy. Cell Res..

[8] Bally APR, Austin JW, Boss JM (2016). Genetic and epigenetic regulation of PD-1 expression. J. Immunol..

[9] Baumeister SH, Freeman GJ, Dranoff G, Sharpe AH (2016). Coinhibitory pathways in immunotherapy for cancer. Annu. Rev. Immunol..

[10] Muenst S, Soysal SD, Tzankov A, Hoeller S (2015). The PD-1/PD-L1 pathway: Biological background and clinical relevance of an emerging treatment target in immunotherapy. Expert Opin. Ther. Targets.

[11] McDermott DF, Atkins MB (2013). PD-1 as a potential target in cancer therapy. Cancer Med..

[12] Ahmadzadeh M (2009). Tumor antigen-specific CD8 T cells infiltrating the tumor express high levels of PD-1 and are functionally impaired. Blood.

[13] Zhang Y, Huang S, Gong D, Qin Y, Shen Q (2010). Programmed death-1 upregulation is correlated with dysfunction of tumor-infiltrating CD8+ T lymphocytes in human non-small cell lung cancer. Cell. Mol. Immunol..

[14] Wong RM (2007). Programmed death-1 blockade enhances expansion and functional capacity of human melanoma antigen-specific CTLs. Int. Immunol..

[15] Sharma P, Allison JP (2015). Immune checkpoint targeting in cancer therapy: Toward combination strategies with curative potential. Cell.

[16] Jiang Y, Chen M, Nie H, Yuan Y (2019). PD-1 and PD-L1 in cancer immunotherapy: Clinical implications and future considerations. Hum. Vaccin. Immunother..

[17] Robert C (2015). Pembrolizumab versus ipilimumab in advanced melanoma. N. Engl. J. Med..

[18] Reck M (2016). Pembrolizumab versus chemotherapy for PD-L1-positive non-small-cell lung cancer. N. Engl. J. Med..

[19] Ferris RL (2016). Nivolumab for recurrent squamous-cell carcinoma of the head and neck. N. Engl. J. Med..

[20] Bellmunt J (2017). Pembrolizumab as second-line therapy for advanced urothelial carcinoma. N. Engl. J. Med..

[21] Schmid P (2018). Atezolizumab and nab-paclitaxel in advanced triple-negative breast cancer. N. Engl. J. Med..

[22] Bergman PJ (2019). Cancer immunotherapies. Vet. Clin. North Am. Small Anim. Pract..

[23] Coy J, Caldwell A, Chow L, Guth A, Dow S (2017). PD-1 expression by canine T cells and functional effects of PD-1 blockade. Vet. Comp. Oncol..

[24] Nemoto Y, Shosu K, Okuda M, Noguchi S, Mizuno T (2018). Development and characterization of monoclonal antibodies against canine PD-1 and PD-L1. Vet. Immunol. Immunopathol..

[25] Maekawa N (2014). Expression of PD-L1 on canine tumor cells and enhancement of IFN-γ production from tumor-infiltrating cells by PD-L1 blockade. PLoS ONE.

[26] Igase M (2020). A pilot clinical study of the therapeutic antibody against canine PD-1 for advanced spontaneous cancers in dogs. Sci. Rep..

[27] Maekawa N (2021). PD-L1 immunohistochemistry for canine cancers and clinical benefit of anti-PD-L1 antibody in dogs with pulmonary metastatic oral malignant melanoma. NPJ Precis. Oncol..

[28] Maekawa N (2017). A canine chimeric monoclonal antibody targeting PD-L1 and its clinical efficacy in canine oral malignant melanoma or undifferentiated sarcoma. Sci. Rep..

[29] Igase M (2022). Long-term survival of dogs with stage 4 oral malignant melanoma treated with anti-canine PD-1 therapeutic antibody: A follow-up case report. Vet. Comp. Oncol..

[30] Folkl A, Wen X, Kuczynski E, Clark ME, Bienzle D (2010). Feline programmed death and its ligand: Characterization and changes with feline immunodeficiency virus infection. Vet. Immunol. Immunopathol..

[31] Harun MSR (2013). Transcriptional profiling of feline infectious peritonitis virus infection in CRFK cells and in PBMCs from FIP diagnosed cats. Virol. J..

[32] Nascimento C (2020). Serum PD-1/PD-L1 levels, tumor expression and PD-L1 somatic mutations in HER2-positive and triple negative normal-like feline mammary carcinoma subtypes. Cancers.

[33] Nascimento C, Gameiro A, Correia J, Ferreira J, Ferreira F (2022). The landscape of tumor-infiltrating immune cells in feline mammary carcinoma: Pathological and clinical implications. Cells.

[34] Agata Y (1996). Expression of the PD-1 antigen on the surface of stimulated mouse T and B lymphocytes. Int. Immunol..

[35] Yamazaki T (2002). Expression of programmed death 1 ligands by murine T cells and APC. J. Immunol..

[36] Liu K (2020). N-glycosylation of PD-1 promotes binding of camrelizumab. EMBO Rep..

[37] Li C-W (2016). Glycosylation and stabilization of programmed death ligand-1 suppresses T-cell activity. Nat. Commun..

[38] Sun L (2020). Targeting glycosylated PD-1 induces potent antitumor immunity. Cancer Res..

[39] Francisco LM, Sage PT, Sharpe AH (2010). The PD-1 pathway in tolerance and autoimmunity. Immunol. Rev..

[40] Ikebuchi R (2013). Blockade of bovine PD-1 increases T cell function and inhibits bovine leukemia virus expression in B cells in vitro. Vet. Res..

[41] Latchman Y (2001). PD-L2 is a second ligand for PD-1 and inhibits T cell activation. Nat. Immunol..

[42] Sugiura D (2019). Restriction of PD-1 function by cis-PD-L1/CD80 interactions is required for optimal T cell responses. Science.

[43] Umehashi M (2002). Post-exposure treatment of cats with mouse-cat chimeric antibodies against feline herpesvirus type 1 and feline calicivirus. J. Vet. Med. Sci..

[44] Gearing DP (2016). In vitro and in vivo characterization of a fully felinized therapeutic anti-nerve growth factor monoclonal antibody for the treatment of pain in cats. J. Vet. Intern. Med..

[45] Strietzel CJ (2014). In vitro functional characterization of feline IgGs. Vet. Immunol. Immunopathol..

[46] Morita S, Kojima T, Kitamura T (2000). Plat-E: An efficient and stable system for transient packaging of retroviruses. Gene Ther..

[47] Jainchill JL, Aaronson SA, Todaro GJ (1969). Murine sarcoma and leukemia viruses: assay using clonal lines of contact-inhibited mouse cells. J. Virol..

[48] Crandell RA, Fabricant CG, Nelson-Rees WA (1973). Development, characterization, and viral susceptibility of a feline (Felis catus) renal cell line (CRFK). In Vitro.

[49] Pedersen NC, Boyle JF, Floyd K (1981). Infection studies in kittens, using feline infectious peritonitis virus propagated in cell culture. Am. J. Vet. Res..

[50] Snyder HW, Hardy WD, Zuckerman EE, Fleissner E (1978). Characterisation of a tumour-specific antigen on the surface of feline lymphosarcoma cells. Nature.

[51] Dunn KJ, Yuan CC, Blair DG (1993). A phenotypic host range alteration determines RD114 virus restriction in feline embryonic cells. J. Virol..

[52] Miura T (1987). Structural abnormality and over-expression of the myc gene in feline leukemias. Int. J. Cancer.

[53] Mochizuki H (2011). Establishment of a novel feline leukemia virus (FeLV)-negative B-cell cell line from a cat with B-cell lymphoma. Vet. Immunol. Immunopathol..

[54] Yamamoto JK (1991). Development of IL-2-independent feline lymphoid cell lines chronically infected with feline immunodeficiency virus: Importance for diagnostic reagents and vaccines. Intervirology.

[55] Hohdatsu T, Hirabayashi H, Motokawa K, Koyama H (1996). Comparative study of the cell tropism of feline immunodeficiency virus isolates of subtypes A, B and D classified on the basis of the env gene V3–V5 sequence. J. Gen. Virol..

[56] Uyama R (2005). Establishment and characterization of eight feline mammary adenocarcinoma cell lines. J. Vet. Med. Sci..

[57] Fujino Y (2004). Characterization of a newly established nonproducer lymphoma cell line for feline leukemia virus. Vet. Immunol. Immunopathol..

[58] Maekawa N (2023). Molecular characterization of feline immune checkpoint molecules and establishment of PD-L1 immunohistochemistry for feline tumors. PLoS ONE.

[59] Mizuno T, Suzuki R, Umeki S, Okuda M (2009). Crossreactivity of antibodies to canine CD25 and Foxp3 and identification of canine CD4+CD25 +Foxp3+ cells in canine peripheral blood. J. Vet. Med. Sci..

[60] Sakai O, Ogino S, Tsukui T, Igase M, Mizuno T (2021). Development of a monoclonal antibody for the detection of anti-canine CD20 chimeric antigen receptor expression on canine CD20 chimeric antigen receptor-transduced T cells. J. Vet. Med. Sci..

