# Supplementary figures and images for: Development of anti-feline PD-1 antibody and its functional analysis
Source: Sci Rep. 2023 Apr 24;13:6420. doi: 10.1038/s41598-023-31543-6 (PMC10126011; doi:10.1038/s41598-023-31543-6)

# Supplement Figure 1

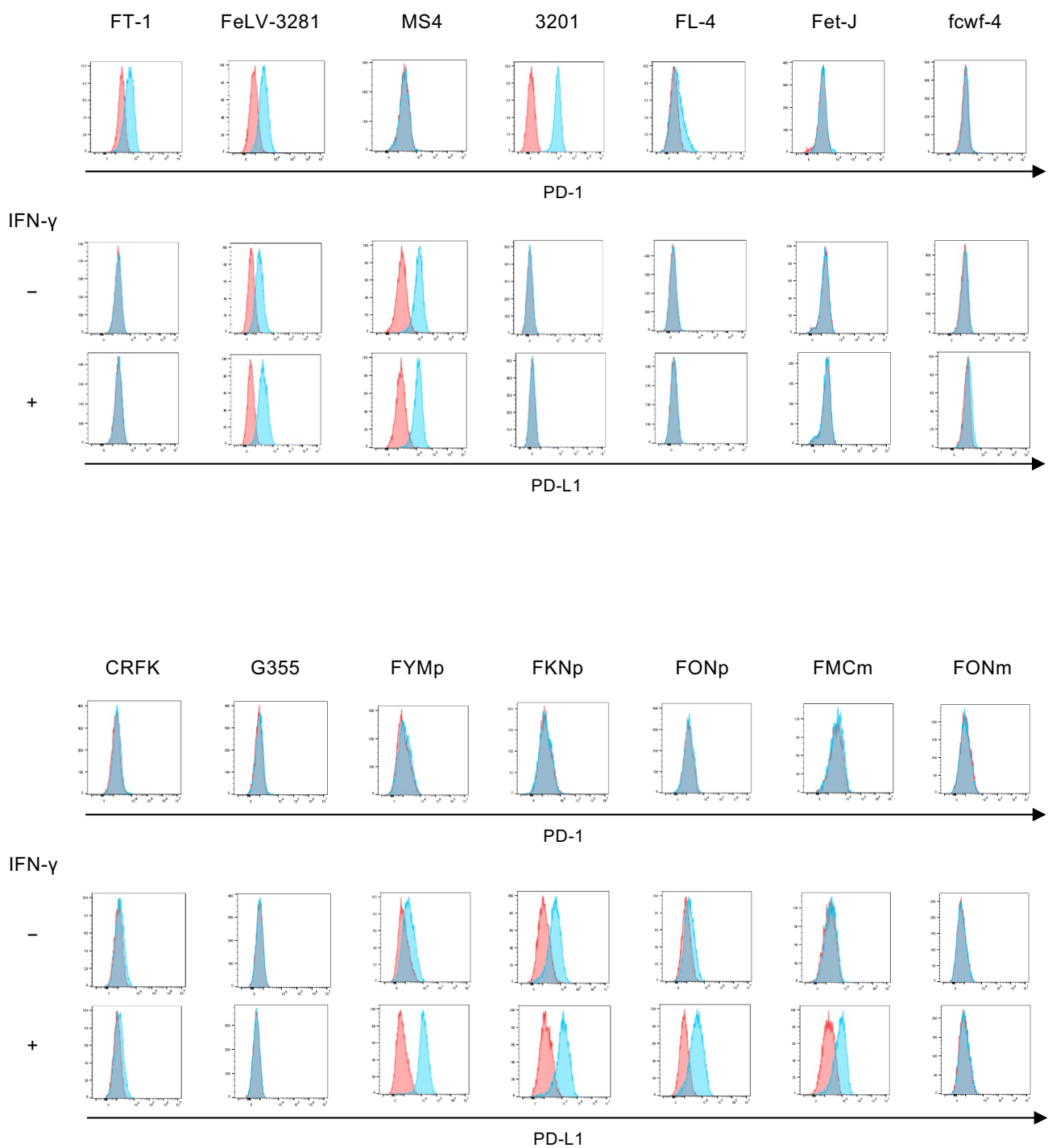

# Supplement Figures for Figure 1A and 2A

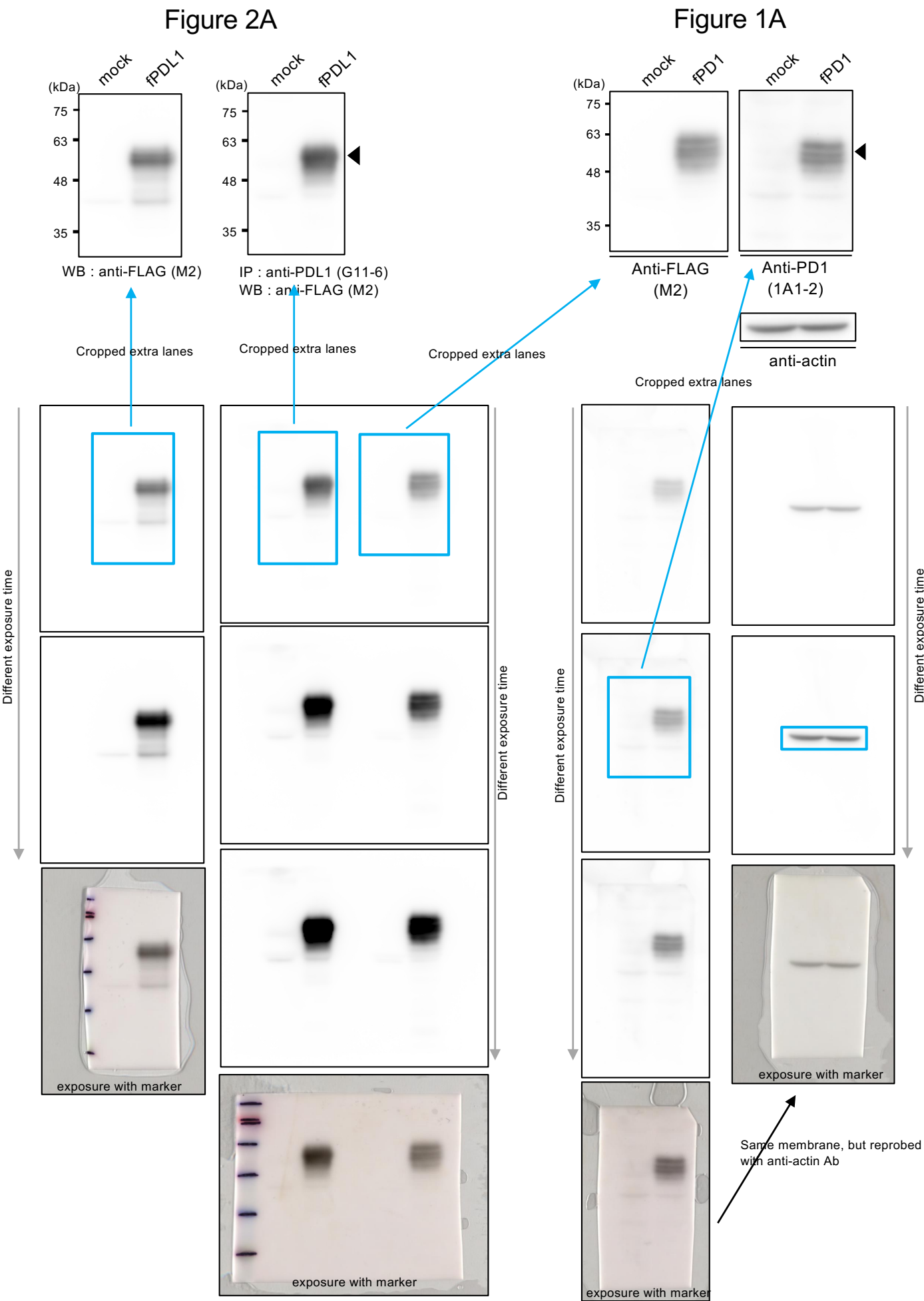

# Supplement Figure for Figure 3A

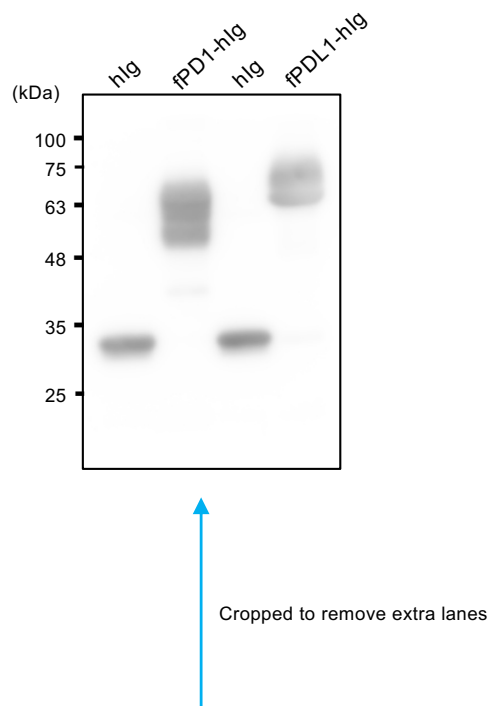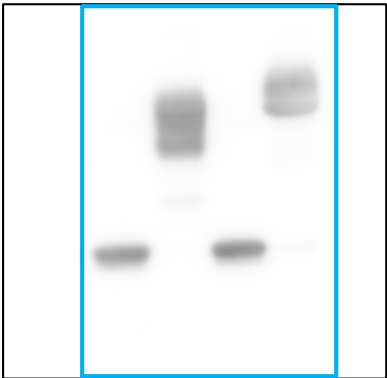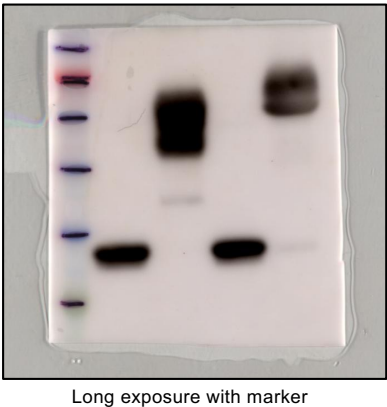

Supplement: Supplementary file 1 — Supplementary Figures. [file 41598_2023_31543_MOESM1_ESM.pdf]
